# A practical checklist for return of results from genomic research in the European context

**DOI:** 10.1038/s41431-023-01328-6

**Published:** 2023-03-22

**Authors:** Danya F. Vears, Nina Hallowell, Heidi Beate Bentzen, Bridget Ellul, Therese Haugdahl Nøst, Angeliki Kerasidou, Shona M. Kerr, Michaela Th. Mayrhofer, Signe Mežinska, Elizabeth Ormondroyd, Berge Solberg, Birgitte Wirum Sand, Isabelle Budin-Ljøsne

**Affiliations:** 1 Biomedical Ethics Research Group, Murdoch Children’s Research Institute, Parkville, VIC 3052 Australia; 2 University of Melbourne, Parkville, VIC 3052 Australia; 3 Centre for Biomedical Ethics and Law, KU Leuven, Leuven, 3000 Belgium; 4 Uehiro Centre for Practical Ethics, University of Oxford, Oxford, OX3 7RF UK; 5 Ethox Centre and Wellcome Centre for Ethics and Humanities, Nuffield department of Population Health, University of Oxford, Oxford, OX3 7RF UK; 6 Centre for Medical Ethics, Faculty of Medicine, University of Oslo, Oslo, Norway; 7 Centre for Molecular Medicine and Biobanking, University of Malta, Msida, Malta; 8 Department of Community Medicine, Faculty of Health Sciences, UiT The Arctic University of Norway, N-9037 Tromsø, Norway; 9 K. G. Jebsen Center for Genetic Epidemiology, Department of Public Health and Nursing, Faculty of Medicine and Health, NTNU, Norwegian University of Science and Technology, N- 7491 Trondheim, Norway; 10 MRC Human Genetics Unit, Institute of Genetics and Cancer, University of Edinburgh, Western General Hospital, Edinburgh, EH4 2XU UK; 11 BBMRI-ERIC, Neue Stiftingtalstrasse 2/B/6, 8010 Graz, Austria; 12 Institute of Clinical and Preventive Medicine, University of Latvia, Riga, Latvia; 13 Radcliffe Department of Medicine, NIHR Oxford Biomedical Research Centre United Kingdom, University of Oxford, Oxford, UK; 14 Department of Public Health and Nursing, The Norwegian University of Science and Technology (NTNU), Trondheim, Norway; 15 Norwegian Institute of Public Health, Legal department, Oslo, Norway; 16 Division of Climate and Environmental Health, Department of Food Safety, Norwegian Institute of Public Health, Oslo, Norway

**Keywords:** Ethics, Genetics research

## Abstract

An increasing number of European research projects return, or plan to return, individual genomic research results (IRR) to participants. While data access is a data subject’s right under the General Data Protection Regulation (GDPR), and many legal and ethical guidelines allow or require participants to receive personal data generated in research, the practice of returning results is not straightforward and raises several practical and ethical issues. Existing guidelines focusing on return of IRR are mostly project-specific, only discuss which results to return, or were developed outside Europe. To address this gap, we analysed existing normative documents identified online using inductive content analysis. We used this analysis to develop a checklist of steps to assist European researchers considering whether to return IRR to participants. We then sought feedback on the checklist from an interdisciplinary panel of European experts (clinicians, clinical researchers, population-based researchers, biobank managers, ethicists, lawyers and policy makers) to refine the checklist. The checklist outlines seven major components researchers should consider when determining whether, and how, to return results to adult research participants: 1) Decide which results to return; 2) Develop a plan for return of results; 3) Obtain participant informed consent; 4) Collect and analyse data; 5) Confirm results; 6) Disclose research results; 7) Follow-up and monitor. Our checklist provides a clear outline of the steps European researchers can follow to develop ethical and sustainable result return pathways within their own research projects. Further legal analysis is required to ensure this checklist complies with relevant domestic laws.

## Introduction

Increasing numbers of European research projects return, or plan to return, individual genomic research results (IRR), (i.e., findings from clinical research or population-based studies that relate to a single individual) to participants [1–5]. IRR might be study-specific results (SSR) relating to the project’s overarching research question(s), secondary findings (SF; actively sought variants associated with conditions or traits unrelated to the research question), or unsolicited findings (UF; incidentally identified disease-causing variants unrelated to the research question)^1^ [6]. Most projects returning results focus on health-related and actionable IRR (those that can lead to surveillance, prevention, or treatment) as this is considered an ethical priority [7].

The return of IRR is, in principle, supported by diverse stakeholders [8–10]. A recent systematic review of 221 empirical articles, encompassing 118,874 individuals across 20 countries (the majority from the USA) identified interest in receiving IRR was high across research participants, patients, and publics, ranging from 47.6–100% [9]. Health professionals, researchers, and institutional review board members are generally more cautious about returning results than participants, patients, and publics were about receiving them [9, 10]. All stakeholders prioritised RoR that could lead to surveillance, prevention and/or treatment. Professionals raised concerns, including difficulties obtaining informed consent, lack of time and resources, possible overdiagnosis, clinical follow-up, and potential for psychological harm [10–17].

**Fig. 1:**
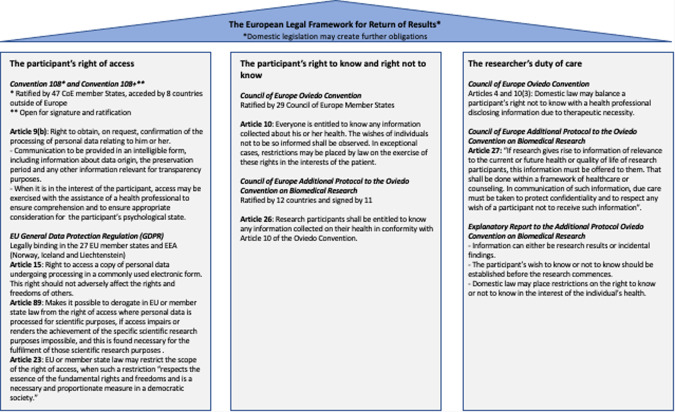
European legal framework for return of results. The three principles underpinning the European framework regarding return of results (the participant’s right of access, the participant’s right to know and right not to know, and the researcher’s duty of care) are articulated here drawing on the four relevant legally binding instruments.

Stakeholders’ views toward return of results (RoR) [8–10] reflect those within the European legal framework, which is underpinned by three principles (the participant’s right of access, the participant’s right to know and right not to know, and the researcher’s duty of care), articulated in four legally binding instruments (Fig. 1) [18–22]. In the European Economic Area (EEA), research participants have a right of access to their health data upon request [18–20] and, in most EEA countries, also rights to know and not know any information collected about their health [21, 22]. In some EEA countries, researchers have a duty of care to offer participants any information of relevance to their current or future health or quality of life [21, 22] and preferences must be ascertained at study outset [23]. There may be domestic legislation in place creating further obligations; e.g., Italy’s genetic data privacy law offers participants choices about RoR, such as an opt-out (right not to know), whereas Spain’s biomedical research law requires researchers to override a participant’s wish not to know when serious harm to the participant or their relatives can be avoided [24]. Article 89 of the General Data Protection Regulation (GDPR) makes it possible to derogate from the participants’ right of access in EU or member state law where personal data is processed for scientific research purposes, if the right of access is likely to render impossible or seriously impair the achievement of the specific scientific research purposes, and derogation from the right of access is necessary for the fulfilment of those scientific research purposes. In such case, appropriate safeguards, including technical and organizational measures, must be in place.

Despite long standing ethical debate [23, 25], there is now broader acceptance that, in addition to the law, there are many ethical and pragmatic reasons to return clinically actionable IRR. While it is generally agreed that participants have ethical, and in several countries legal, rights to receive personal research data, and that researchers may have an obligation to return it, depending on the nature of the relationship established with data contributors [26], the practice of RoR raises several practical issues.

Returning IRR requires researchers to consider the scope of findings to be returned, the strength of evidence for clinical utility, how to organize the informed consent process [27, 28], gather the necessary expertise and resources to interpret variants and return results [29], and establish logistical infrastructure to support the return process [30], and potential follow-up [31]. Recent guidelines provide an overview of steps needed to plan for, and organise, the RoR process [32–34]. However, most of these are project-specific [32], focus on which results to return [33] rather than how to organize the RoR process, or were not developed specifically for the European context [34]. The European Society of Human Genetics (ESHG) recommendations for the clinical use of whole genome sequencing suggest caution in the return of UF [35]. These guidelines were not designed to apply to research settings and are now nearly a decade old in a field where technology and genomic data generation are progressing rapidly. Recent ESHG guidance does not recommend active searching for SF in a clinical setting [36].

In 2015, experts participating in the European COST Action “Citizen’s Health through public-private Initiatives: Public health, Market and Ethical perspectives” (CHIP ME) IS1303 [37] network, of which I.B.L and D.F.V were members, recommended development of European harmonized, equitable, scientifically sound, and socially robust guidelines for return of genetic/genomic IRR [38]. Such guidelines would support European researchers and/or biobanks planning and managing RoR, irrespective of differences at micro (research laboratory/group), meso (university/institute/biobank), and macro (region/country) levels. It was argued guidelines should be applicable to both disease-focused and population-based research. Others too have suggested that although it will not always be possible for international collaborations to overcome discrepancies by harmonising their RoR policies and tools, collaborations may want to design RoR processes that still allow for decisions to be made locally [24].

To address this need for practical guidance, and growing interest in RoR, an analysis of normative documents and expert consensus were used to develop a checklist of steps to assist researchers and/or biobanks in Europe considering returning IRR to adult participants to: a) decide whether RoR is appropriate, feasible, and sustainable for their project, b) develop a RoR plan, and c) implement a RoR pathway.

## Methodology

The initial drafted checklist was based on existing normative documents that provided guidance on returning IRR. To identify these, a Google search was conducted in October 2020 using the following terms: [(guidance OR guideline OR policy OR recommendation) AND (return OR feedback OR individual OR genetic OR research results OR incidental findings)]. Documents underwent full-text review and were included if they were a) regional, national, or international guidelines providing practical information about steps for RoR; b) publicly available and published in English in the preceding 10 years (based on technological advancements made in this time); and c) for clinical research and/or population/biobank research. They did not need to discuss genetic/genomic research specifically nor be academic publications. Documents were excluded if they only discussed the pros and cons of feedback, only provided a list of genetic variants to return, focused on single aspects of RoR (e.g., feedback to relatives, children, deceased), or were specific to research areas (e.g., epigenetics, psychiatry, imaging) or disease (e.g., cancer).

Documents were read by a member of the research team (I.B.L.) and text that provided information about the steps that needed to be taken or aspects that require consideration to return IRR was extracted into a spreadsheet. Inductive content analysis was used to analyse the data [39]. Data were categorised into broad content categories corresponding to stages within the research process (e.g., planning of research project, informed consent, etc.). Each point within the extracted text was coded (e.g., participants should be offered the option to receive results). Coding of all points was performed by two researchers independently (D.F.V. and I.B.L.). Similar codes were grouped together (D.F.V.) to form subcategories within the broad content categories (e.g., ‘whether results will be returned’ under the ‘develop a plan’ category), then checked and refined (N.H.). All the broad categories and subcategories formed the basis for the first draft of the checklist. As the documents included pertained to RoR in adults, rather than minors, we limited the checklist to RoR in adult research populations.

To gauge the checklist’s relevance to the European context, we sought feedback from a panel of European interdisciplinary experts in RoR-related fields across both clinical research and population-based settings in a seven-hour digital workshop over three days in June 2021. Expert panel members (EPs) were recruited from professional networks of the study team (D.F.V., N.H., I.B.L.), including the COST Action CHIP ME and European Society of Human Genetics, and projects known by the study team to have experience of either returning or planning to return IRR. EPs (*n* = 27) included: clinicians, clinical researchers, population-based researchers, biobank managers, bioethicists, lawyers and policy makers. EPs watched presentations about several population-based research initiatives that are returning results (HUNT 4, FinHealth P5, Estonia Biobank, 100,000 Genome project), the legal frameworks pertaining to RoR (H.B.B.), and stakeholder perspectives (D.F.V.). The draft checklist was presented and feedback was obtained from EPs using structured discussion facilitated by I.B.L., D.F.V. and N.H. A range of views were presented in the workshop; we report herein the majority consensus (>75% agreement). Discussions were recorded, transcribed, coded and categorised akin to the normative documents. New categories or subcategories arising and agreed upon in the discussion were added to the checklist; none were excluded (I.B.L., D.F.V. and N.H.).

## Results

Of the 34 normative documents identified, six met our inclusion criteria, two of which were from Europe/UK (Table 1). The checklist provides an overview of the procedural steps European researchers and/or biobanks should consider to enable RoR to consenting adult research participants, from conception of a project to its full realization, including the production and disclosure of clinically actionable IRR. The checklist applies to both population-based research and clinical research projects. Below we describe the seven steps of the checklist (Table 2), which are intended to guide researchers considering RoR as they 1) decide which results to return; 2) develop a plan for return of results; 3) obtain participant informed consent; 4) collect and analyse data; 5) confirm results; 6) disclose research results; and 7) follow-up and monitor participants and processes.

**Table 1:** The six normative documents that met inclusion criteria.

Title	Year	Organization	Country	Genomic-specific	Reference number
National Statement on Ethical Conduct in Human Research (Chapter 3 - genomic research)	2018	Australian Govt/National Health and Medical Research Council	Australia	Yes	[40]
Consensus report: Returning individual research results to participants	2018	National Academies Science Engineering Medicine	USA	No	[43]
BBMRI-ERIC Guide to the detection, management and communication of incidental findings for biobanks in BBMRI-NL	2017	BBMRI-ERIC	Europe	No	[33]
MRCT Center Return of Individual Results to Participants Recommendations Document	2017	Multi-Regional Clinical Trials Center of Brigham and Women’s Hospital and Harvard	USA	No	[42]
Framework on the feedback of health-related findings in research	2014	Medical Research Council/Wellcome Trust	UK	No	[41]
ANTICIPATE and COMMUNICATE Ethical Management of Incidental and Secondary Findings in the Clinical, Research, and DTC Contexts	2013	Presidential Commission for the Study of Bioethical Issues	USA	No	[46]

*RoR* return of results, *IRR* individual research results, *UKAS* United Kingdom Accreditation Service, *LMI* low- and middle-income countries, *REC* research ethics committee, *SF* secondary findings, *UF* unsolicited findings.

**Table 2:** Checklist of the steps required for the return of IRR.

1. *Decide which IRR to return*		
**Consider the nature of IRR**	**Consider the context of result return**	**Consider the practicalities of RoR**
□ Clinical study vs population-based research □ Clinical utility/health significance and medical actionability □ Urgency and severity of the results □ Potential impact on participants (eg. psychological impact, presence of an intervention) □ Benefits and harms of RoR □ Analytical and scientific validity, incl. positive/predictive value, and false positive rates □ Limitations on test validity and interpretation □ Quality and external review process (e.g., UKAS certified laboratory) □ Involve clinical expertise in decisions	□ Participant needs, preferences, values □ Age of study population, if relevant. □ Potential return to minor participants becoming adults □ Vulnerability of study population, ability to make decisions about RoR, and access to health care (e.g., in LMI countries) □ National good practice guidelines □ Country-specific resources to support RoR □ Obligations for RoR beyond research scope/after study completion □ Obligations to search for SF	□ Disclosure pathway and timeframe □ Clarifying role of treating physician in RoR □ Privacy and confidentiality issues □ Possibility to recontact participants □ Infrastructure for data storage and future analysis □ Sustainability of resources and feasibility of RoR □ Logistical requirements for RoR and training of staff □ Research aims that may potentially be compromised if RoR
2. *Develop a plan for return of results*		
Include:		
□ Types of results to be generated (primary, secondary, unsolicited findings) and how (e.g., techniques to be used)		
□ Types of results to be returned		
□ Justifications for returning or not returning results		
□ How informed consent will be obtained		
□ Processes for clinical assessment and validation of results, (if applicable)		
□ Reference to relevant institutional policies relating to RoR		
□ Timing, frequency, and duration of return		
□ Mechanisms to locate expertise for RoR		
□ Mechanisms to handle potential requests for RoR		
□ Mechanisms to return results to participants and by whom		
□ Possible psychological effects of RoR		
□ Mechanisms to secure safety of participants		
□ Whether approval from data access committee will be needed to return results and role of data protection officers		
□ The responsibilities of researchers after disclosure		
□ The potential role of associated biobanks in RoR process		
□ Budget, resources, and infrastructure to support RoR		
□ Include plan in funding application, scientific review, and ethics application to REC		
□ Factor in time for REC amendment if required		
3. *Obtain participant consent to return results*		
*Provide information in the informed consent about:* *Background and rationale*		
□ Study purpose, including uses of samples for genetic research		
□ Distinction between research and clinical care		
□ Likelihood of RoR, limitations of RoR and rationale for RoR		
□ Types of results that may be fed back (including unanticipated and unsolicited findings)		
*Management of RoR*		
□ How results will be disclosed, by whom, when, and frequency of RoR		
□ Conditions under which urgent results may be fed back		
□ Procedure for collecting the participant’s consent to communicate results to his/her health care provider and/or place results in medical records		
□ Possibility that consent may be sought at multiple time points to enable participants to reassess decisions regarding RoR		
□ Possibility for participants to request results		
□ Possibility for participants to be re-contacted and procedure for consent to re-contact		
*Implications for participants*		
□ Potential risks of RoR, e.g., physical, psychological, unanticipated risks		
□ Potential benefits of RoR, e.g., commercial profit, access to tests, preventative treatments		
□ Possibility of accessing clinical/genetic counselling services		
□ Possibility of follow-up, e.g., investigations and interventions		
□ Potential implications of RoR for the participants’ relatives		
□ Possibility of sharing results with relatives in case of participant death		
*Privacy and confidentiality issues*		
□ Procedures for privacy and confidentiality protection and data de-identification		
□ Risks of potential re-identification of genetic information		
□ Limits of confidentiality and risks of confidentiality breaches		
□ Plans for data security and sample storage and management		
□ Country specific discrimination laws		
*Secondary and third-part use*		
□ Data access by other researchers for secondary research		
□ Possibility to choose whether to allow secondary use of data		
□ Data access by third parties		
□ Role of industry		
□ Data sharing on open access platforms		
*Study contacts for questions and additional information*		
*Requirements of biobank/registry (if applicable)*		
4. *Collect and analyse data*		
□ Use tests necessary to answer the research question		
□ Include clinicians with expertise in handling clinically significant genomic findings		
□ Inform researchers about procedures to deal with UF		
□ Search for SF if easy to carry out the additional analysis		
5. *Confirm results*		
□ Establish utility and accuracy of test		
□ Ally with experts to validate results, e.g., accredited lab/repeated testing multidisciplinary teams		
□ Involve participant to confirm analytical validity (e.g., to collect additional sample for result validation) if necessary		
6. *Disclose research results*		
□ Identify results provider for IRR (e.g., primary health care provider) or consider direct return to participant		
□ Consider the use of emerging technologies for IRR		
□ Check skills and qualifications of results provider (e.g., skills documentation)		
□ Ensure participant is promptly informed about IRR		
□ Tailor information to the needs and preferences of participants		
□ Inform participants about meaning and implications of results, and level of uncertainty and provide a written summary of the results		
□ Discuss implications of RoR for the participant’s relatives		
7. *Follow-up and monitor*		
□ Consider avenues for follow-up (e.g., medical specialists/clinical services) and provision of clinical referral, and inform participants		
□ Monitor the effects of communicating IRR		
□ Evaluate the management of IRR policy		

### 1. Decide which results to return

#### Consider the nature of IRR

In addition to the legal requirements for RoR in particular jurisdictions (Fig. 1), decisions regarding which IRR to return to participants should include whether the finding is an SSR, which may depend on whether the study is clinical research or population-based, its potential health or clinical significance [33, 40, 41], its medical actionability [40, 41], and the associated condition [42]. Guidelines suggest clinical experts assist with these determinations [33]. Researchers should consider the potential benefits and harms associated with RoR [41, 43], including participant’s best interests [41], and the potential impact on the participant, including the impact of uncertainty, and the presence/absence of and ability to access an intervention [42]. Countries signatory to the Oviedo Convention’s Additional Protocol on Biomedical Research, have a legal duty of care, which includes returning research information relevant to the current or future health or quality of life, but leaves the interpretation of “care” to the evaluation of researchers and research ethics committees [23, 43]. The findings’ clinical actionability, and analytical and scientific validity should be considered (e.g., positive predictive value, false positive rates) [40, 41, 43], along with limitations on test validity, analysis quality and laboratory accreditation [43] and whether the result will be validated in a clinical laboratory.

Resources should be consulted to support decisions about returning UF (e.g., scientific literature [33], online resources (e.g., ClinVar) [44], internationally accepted guidelines for variant interpretation, (e.g., the American College of Genetics and Genomics; ACMG) [45]). Either a predetermined list or protocol [32], (e.g., the ACMG gene list) [34], or returning exploratory results on a case-by-case basis may be appropriate [42]. Researchers should consider involving clinical expertise in such decisions [23, 32, 43].

#### Consider context of result return

Researchers should consider the RoR context [22], including study population vulnerability and access to appropriate healthcare [41]. Participant age may also be relevant; EPs mentioned examples where research participants, minors at the time of (parental) consent, reach the age of majority. Participant needs, preferences and values should be incorporated into RoR decision-making [43]. Researchers should follow country-specific good practice guidelines for UF (if they exist) [41] and EPs suggested researchers investigate country-specific funding options/resources and relevant laws to support RoR.

The Australian National Health and Medical Research Council (NHMRC) specifies there is no obligation to assess or return findings beyond research scope/study completion [40].

#### Consider the practicalities of RoR

If IRR will be returned, researchers must develop a clear disclosure pathway, subject to participant informed consent [40, 41], and determine how to return results in a reasonable timeframe based on when findings will be identified [41]. Consideration should be given to privacy and confidentiality issues relating to data handling, such as the impact of linking identifiers to the samples, collection of excess sample, and family member involvement [42]. Non-European guidelines suggest return decisions regarding both SSR and UF should assess the benefit of return against availability of resources, feasibility and sustainability, including staff training for RoR [41–43]. EPs urged researchers to consider identifying appropriate healthcare professionals (e.g., specialist physicians, genetic counsellors), involving them in protocol development, and clarifying the precise role of primary care or specialist healthcare professionals (where relevant) in the disclosure pathway [22]. Logistical requirements requiring consideration include whether researchers (or relevant healthcare teams) can recontact participants [41], whether future analysis is feasible and if so, availability of infrastructure for data storage and reanalysis [40].

Researchers should assess whether RoR will compromise the research aims [46]. One non-European guideline suggests IRR should be offered at study completion if feasible and study integrity is not compromised [42].

### 2. Develop a plan for return of results

Most guidelines stressed the importance of developing an RoR plan when designing the study [40–43]. This plan should cover a range of elements (Table 2) including clear descriptions of how (i.e., the intended techniques) [40], and the types of results likely to be generated, including SSR, UF and SF [33, 40–42]. EPs emphasised the importance of distinguishing between these types of results and drawing clear distinctions between research and clinical results, as highlighted in the NHMRC National Statement [40]. EPs suggested researchers consider whether separate protocols are needed for return of SSR versus SF/UF. The plan should describe whether, and which types of results will be reported [33, 40, 41, 43]. The rationale for return, which results will be returned at which time points, who will be responsible for making such judgments, return processes for both SSR and UF, and the research team’s responsibilities after result disclosure [46] should be provided [40, 43, 46].

RoR plans should describe how participants’ informed consent and RoR preferences will be sought, and how researchers intend to respond to RoR requests [43], although in the EEA, this is regulated in law. The plan should include whether findings will be validated in a clinical laboratory, the processes for this, whether additional resources or expertise are required [40, 41], how this expertise will be sourced (if not already contained within the research team), and any distinction between analytical and clinical validity [40].

The plan should describe the budget, infrastructure and resources required [43]; both EPs and a US document emphasised ensuring adequate resources to support RoR [43]. They suggest the RoR plan should be described in project funding proposals and that funding bodies incorporate RoR funding into the project budget [43], although EPs recognised this may be challenging. EPs stressed the importance of mentioning whether approval from Data Access Committees will be needed, although they cannot request additional requirements above those imposed by law. The plan should mention any biobanks associated with the study and their role in the RoR process [40], as well as relevant institutional policies [43].

Researchers should seek approval for their project, including the RoR plan, from a Research Ethics Committee (REC) [40, 42, 43, 46], and factor in time for REC changes, which may alter the RoR plan. To ensure sufficient funds for RoR activities, applications for funding should occur prior to seeking REC approval; in some countries, approved funding of projects is a pre-condition for REC approval.

### 3. Obtain participant informed consent to return of results

When designing the consent process, it is important to ensure the information provided is clear and accessible [41]. Here we focus specifically only on the aspects to be covered in the informed consent (IC) process relating to RoR, not all points required by GDPR. It should discuss the identified through the research (including UF/SF) [33, 41, 46], the likelihood of these occurring [40, 41], which results may be returned to participants and the rationale for return [33, 41–43].

Participants should be informed of the potential risks of receiving results [43], including psychological risks and overdiagnosis, any unanticipated risks [42], and insurance implications. Limitations of identified results should be highlighted [41] as should the fact that variant pathogenicity may be reassessed over time. EPs suggested mentioning the potential benefits to participants’ and blood relatives’ health.

Participants should be informed whether they can choose which types of results they wish to receive [41, 42], including not receiving results (i.e., the right not to know as highlighted by EPs and which may be enshrined in national law in most EEA countries) [41, 43, 46]. Researchers should discuss whether participants will be able to reassess their RoR choices [40]. Participants’ decision about RoR should be respected [40, 46]; researchers should have a plan, which may include consulting their relevant REC and domestic law, if serious actionable results are identified in a participant who chose not to receive them [46].

Researchers should describe how results will be returned [33, 40–43, 46] including whether they will be communicated to participants’ healthcare providers and placed in their medical records [43], and the timeframe and conditions under which results may be returned [40, 43]. They should discuss who will communicate results [40] and the potential implications of the findings [41, 42], such as access to genetic counselling [40, 46], the potential for interventions or follow-up [40, 41], and who will pay for these if healthcare is not free at the point of need [41]. Distinctions between research and clinical care should be made clear to avoid therapeutic misconception [41, 46], as should implications of results for relatives [40] and whether results will be shared with relatives in general or in case of the participant’s death [43].

### 4. Collect and analyse data

Only one guideline addressed data collection and analysis considerations, which was specifically for UF [33]. This European document suggests researchers should use the tests required to answer the research question but that research teams include healthcare professionals with expertise in handling clinically significant genomic findings [33]. The US Presidential Commission states that researchers may get approval but are not obligated to search for SF [46]. Practically, this might only be possible if the research is conducted in a clinical setting, as seen in the 100,000 Genomes Project [47].

### 5. Confirm results

Researchers should consider only returning results once they have been validated in a clinical laboratory and the utility of the result established [40]. EPs suggested clinical validation should be done by an accredited laboratory using evidence-based standards (e.g., ACMG) [33, 40, 41, 45]. EPs highlighted the importance of multidisciplinary teams to ensure expert laboratory and clinical input and reporting consistency. One document suggests it may be necessary to involve the participant when confirming analytical validity, (e.g., to collect a second sample for result validation) [41].

### 6. Disclose research results

Researchers should determine who is responsible for, and will be actively involved in RoR, and how and when this will occur. Guidelines suggest returning IRR should ideally be the responsibility of either an appropriate clinical service or the participant’s clinician in discussion with the research team [40]. EPs stressed it is important to ensure the person providing results is appropriately qualified and certified to country or institution standards [33, 41]. Participants should be informed about the meaning and implications of RoR for themselves and family members [40, 43].

The pathway for returning IRR should be clear and situation-specific [41, 42] and results should be provided in a timely manner [33]. Researchers should consider how results will be returned: in person, by telephone or video conference, via online platform, by a confidential letter, or by a combination of these strategies. If REC approval is obtained to return results directly to participants, researchers should consider engaging with emerging technologies, such as IT-based solutions, to best tailor the information to participants’ needs and preferences [33, 43]. One guideline suggests participants should always be provided with a written summary of the results, regardless of other forms of communication used [43].

### 7. Follow-up and monitor

Several guidelines state researchers should consider how RoR will allow participants to access clinical follow-up and inform them about this option where appropriate [41, 43]. One guideline, which limits its guidance to UF, suggests researchers should ally with medical specialists to allow clinical follow-up and offer to help participants with this process [33]. EPs suggested researchers should develop a pathway for participants to obtain a clinical referral when needed. One guideline suggests researchers should monitor the effects of communicating UF and evaluate the management of UF policy within the research project [33].

## Discussion

We used a mixed methods approach, drawing on existing guidelines and expert perspectives, to develop a practical checklist for researchers and/or biobanks considering RoR to participants. The European legal framework for returning results to research participants includes four legally binding instruments [18–22]. Although they constitute a minimum threshold that must be met to fulfil legal obligations in pan-European projects, these instruments are not ratified in all European countries. As article 89 of the GDPR shows, derogations and additional requirements to these instruments may be given in domestic legislation creating further obligations. Such diversity in international, regional and national laws/policies raises challenges for research combining datasets across multiple jurisdictions [24]. While the law pertaining to RoR may be the same in both population-based research and clinical research contexts, we acknowledge the ethical obligation to return results may vary [27].

When using this checklist, we suggest researchers consider several overarching aspects. First, experiences from previous projects show returning results requires extensive resources to establish appropriate and sustainable infrastructure, obtain the necessary approvals, and assign staff to RoR [48]; these may be difficult to fully assess and/or acquire before the RoR process begins [7, 31]. The suggestion for results to be returned by an appropriate clinical service or the patient’s clinician may be difficult to achieve given the lack of genetics-trained health professionals to meet clinical demand, let alone manage return of research results. Researchers should plan for some flexibility to ensure the cost-effectiveness of RoR [49] and participant education and counselling expenses [48].

Second, researchers should utilise collaborations with clinical experts (e.g., clinical geneticists, genetic counsellors) to develop the RoR plan and return results. Greater clinician support for the return process will increase the perceived utility and clinical usage of results [31].

Third, researchers should be encouraged to share their experiences of the return process, promote best practices through publications, attend conferences to build competence, encourage harmonization of RoR processes across projects and/or biobanks, and share variant data in international databases.

A strength of our approach is that we combined recommendations from existing guidelines with perspectives of European experts across a range of related fields, many of whom have RoR experience. This is important because some existing guidelines were written before much experience had been accumulated. Documents published later than 2020 were excluded from the analysis. However, we note valuable recent contributions to the landscape from Lewis et al. [50] and Willis et al. [51]. In particular, the GA4GH Policy on Clinically Actionable Genomic Research Results supports devising a specific RoR protocol and acquisition of resources for RoR prior to study commencement [50]. Critically, the checklist does not specify a list of genes or specific variants to be returned to participants; this should be determined when study protocols are developed, by a multidisciplinary research team, based on current scientific knowledge and the research context.

Another strength is that utilising European panel members, including legal experts, ensures the checklist is relevant to the European context, as well as more globally. The recommendation to check the three principles articulated in the four legal instruments, as well as country-specific laws [Fig. 1], and a more conservative approach to SF align well with its use in Europe.

While we consider our approach thorough, it is possible some considerations were missed. We encourage researchers to test the checklist by implementing RoR processes into their research projects to identify any gaps. We targeted the checklist to researchers working in research projects/biobanks at their conception, rather than existing studies. Although many of the same considerations pertain when developing RoR processes in existing projects, several aspects, such as obtaining consent, will require consideration.

## Data Availability

The data from the analysis of the normative documents is available from the corresponding author on request.

## Footnotes

1 We refer to all potentially returnable findings as IRR unless the guidelines we are referring to specifically state a particular type of IRR.
